# Elevated effluent potassium concentrations predict the development of postreperfusion hyperkalemia in deceased liver transplantation: a retrospective cohort study

**DOI:** 10.1186/s12871-022-01699-1

**Published:** 2022-05-25

**Authors:** Liang Zhang, Fu-Shan Xue, Ming Tian, Zhi-Jun Zhu

**Affiliations:** 1 Department of Anesthesiology, Beijing Friendship Hospital, Capital Medical University, No. 95 Yong-an Road, Beijing, 100050 China; 2 Division of Liver Transplantation, Department of General Surgery, Beijing Friendship Hospital, Capital Medical University, Beijing, China; 3 Clinical Center for Pediatric Liver Transplantation, Capital Medical University, Beijing, China; 4 Liver Transplantation Center, National Clinical Research Center for Digestive Diseases, Beijing, China

**Keywords:** Hyperkalemia, Potassium, Effluent, Liver transplantation, Reperfusion

## Abstract

**Background:**

Postreperfusion hyperkalemia (PRHK) has garnered increasing attention in regard to deceased liver transplantation (LT), especially for LT using the expanded criteria donor grafts. However, the impact of the effluent potassium (eK^+^) concentration on PRHK has been largely overlooked. We evaluated whether elevated eK^+^ concentrations are associated with PRHK in deceased LT.

**Methods:**

In this single-institution, retrospective cohort study, we included all adults who underwent deceased LT with intraoperative eK^+^ concentration monitoring between November 2016 and December 2018. The eK^+^ concentrations were obtained from the effluent samples collected following a standard portal vein flush. PRHK was defined as any serum potassium (sK^+^) level of > 5.5 mmol/L following reperfusion. Logistic regression was performed to identify predictors for PRHK, and linear regression was used to examine predictors of the maximum percentage increase in the sK^+^ level following reperfusion.

**Results:**

Of the 86 patients who met the inclusion criteria, 54 (62.8%) developed PRHK. Independent predictors for PRHK included greater graft weight (OR 1.283 [95% CI 1.029–1.599] per 100 g, *P* = 0.027), an elevated eK^+^ concentration (OR 1.291 [95% CI 1.068–1.561] per mol/L, *P* = 0.008), and a higher sK^+^ level before reperfusion (OR 4.459 [95% CI 1.543–12.884] per mol/L, *P* = 0.006). An eK^+^ concentration of more than 6.9 mmol/L had a sensitivity of 59.26% and a specificity of 78.12% for predicting PRHK (area under the receiver operating characteristic curve, 0.694). Multiple linear regression analyses indicated that the eK^+^ and sK^+^ levels before reperfusion were significant predictors of the maximum percentage increase in the sK^+^ level following reperfusion. In addition, PRHK was associated with an increased risk of postreperfusion significant arrhythmias, severe postreperfusion syndrome, and postoperative early allograft dysfunction.

**Conclusions:**

This study shows that the eK^+^ concentration could predict the risk of PRHK in deceased LT. Further prospective studies are warranted to clarify these associations.

## Background

Postreperfusion hyperkalemia (PRHK) is a well-known and potentially life-threatening complication during liver transplantation (LT). PRHK may induce severe postreperfusion syndrome (PRS) [1], and its most serious consequence is cardiac arrest [1–7], which is associated with higher intraoperative mortality and poorer graft and patient survival after transplantation [4–7]. Therefore, identifying risk factors for PRHK, especially novel, modifiable predictors, may help clinicians develop targeted preventive strategies.

Several published studies have examined predictive risk factors for PRHK in adult LT. In 2000, Nakasuji and colleagues first demonstrated that the cardiac index, serum lactate levels, and serum potassium (sK^+^) levels during the anhepatic stage are independently associated with the peak sK^+^ levels immediately following reperfusion [8]. One large-sample study in 2007 by Xia et al. [9] found that intraoperative hyperkalemia occurred most frequently in the early reperfusion period, with higher baseline or prereperfusion sK^+^ levels and implantation of donation after cardiac death (DCD) liver grafts as the main contributing factors for PRHK during adult LT. Subsequently, researchers on the same team at the University of California, Los Angeles, determined that the storage age of transfused red blood cells (RBCs) was an independent risk factor for PRHK in adults undergoing LT [10]. More recently, an increasing number of studies have reported the link between the use of DCD, steatotic, and other expanded criteria donor (ECD) liver grafts and PRHK in deceased LT [11–13].

However, most of these predisposing factors are often unmodifiable, and the exact pathophysiological mechanism of PRHK caused by ECD liver grafts has not been fully elucidated. Given that effluent potassium (eK^+^) concentrations following a standard portal vein flush (PVF) are generally elevated during LT using ECD liver grafts [13, 14], we conducted this retrospective cohort study to assess the association between eK^+^ concentrations and the development of PRHK in adult patients undergoing deceased LT.

## Methods

### Patients

The study protocol was approved by the Institutional Review Board of Beijing Friendship Hospital (2020-P2-042–01). Informed consent was waived because of the retrospective nature of this study. This study is reported following Strengthening the Reporting of Observational Studies in Epidemiology (STROBE) guidelines [15]. Eligible participants were adults who underwent deceased LT with intraoperative eK^+^ concentration monitoring at the Beijing Friendship Hospital between November 2016 and December 2018. Exclusion criteria were age < 18 years, hyperkalemia diagnosed prior to reperfusion, no sK^+^ level measurement at one minute following reperfusion (sK^+^_1_), and incomplete data.

### Surgical technique and anesthesia protocol

**Fig. 1:**
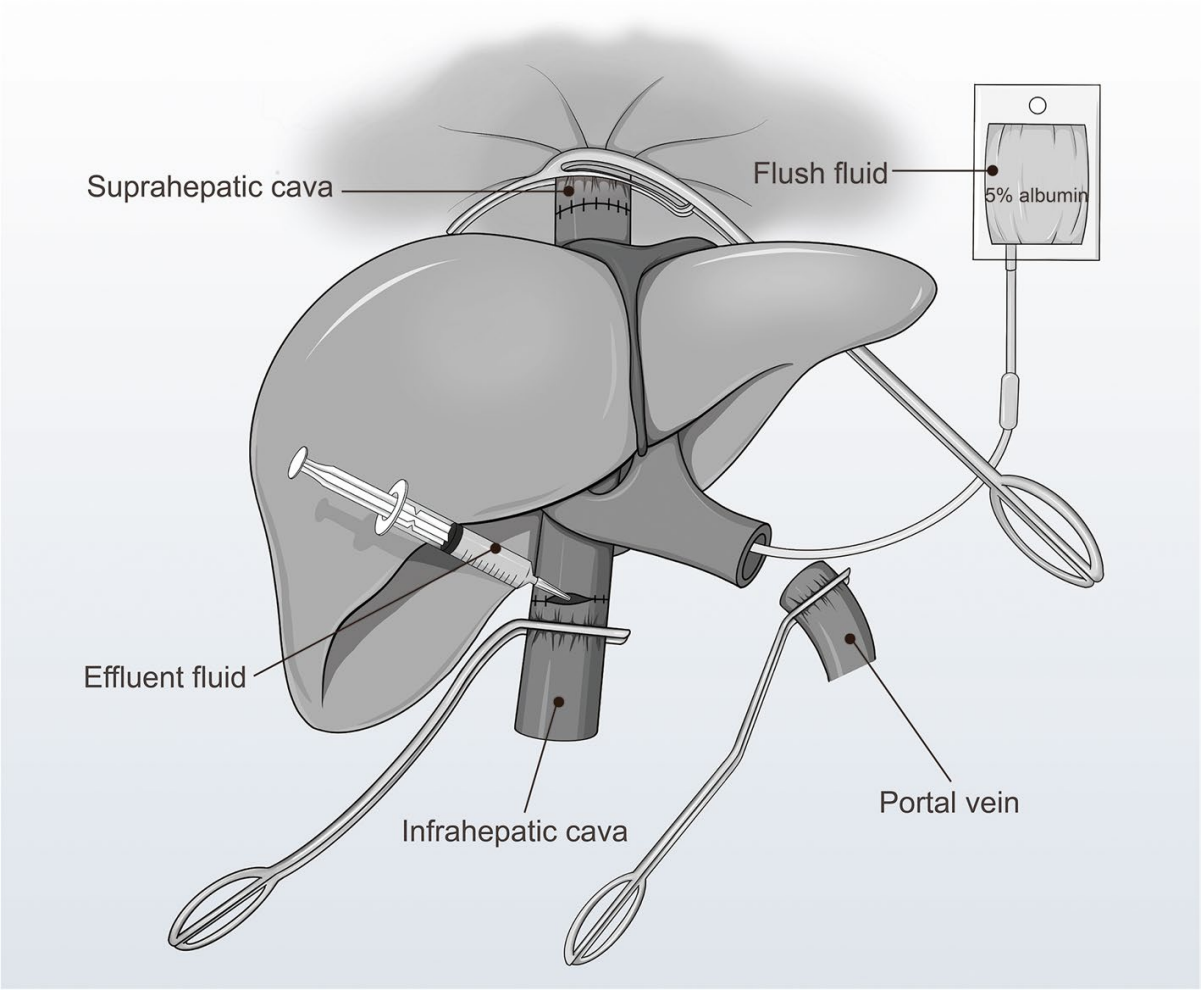
Schematic representation of the sampling site for the eK^+^ measurements following a standard portal vein flush. eK^+^, effluent potassium concentration

The surgical procedures have been described in detail previously [14]. Briefly, liver grafts were implanted using the conventional caval replacement technique without venovenous bypass. Before reperfusion, the sequence of the vascular anastomosis was the suprahepatic vena cava, infrahepatic vena cava, and portal vein. Before the infrahepatic vena cava anastomosis was completed, the graft was rinsed with a 5% albumin solution to flush out the University of Wisconsin (UW) solution. The amount of 5% albumin solution used in our practice was one millilitre per gram of graft weight. Specifically, the eK^+^ concentrations were obtained from the effluent samples collected via the infrahepatic vena cava following standard PVF (Fig. 1) and were measured using a point-of-care blood gas analyzer.

Perioperative anesthetic care was carried out according to the standard of care at our institution [14]. During the study period, intraoperative sK^+^ levels were generally monitored by performing blood gas measurements at the following fixed time points: 1) before incision; 2) immediately before portal vein clamping; 3) immediately before reperfusion; 4) at one minute following reperfusion; 5) at 5 min following reperfusion; 6) at one hour following reperfusion; 7) at 2 h following reperfusion; and 8) at the end of the surgery. Notably, most blood samples were collected from the arterial lines except the blood sample one minute following reperfusion, which was collected from the pulmonary artery catheters.

### Data collection

Our institutional database was used to collect baseline patient characteristics, intraoperative variables, and postoperative outcomes retrospectively.

- Baseline patient characteristics included age, sex, weight, height, primary diagnosis, Child–Pugh score, and Model for End-Stage Liver Disease (MELD) score.
- The intraoperative variables collected included graft weight, graft-to-recipient weight ratio (GRWR), cold ischemia time (CIT), warm ischemia time (WIT), inferior vena cava clamping time, duration of the anhepatic phase, prereperfusion amount of RBCs transfused, presence of an ECD liver graft (DCD donors, donor age of > 65 years, a sodium level of > 155 mmol/L, aspartate aminotransferase or alanine aminotransferase levels of > 100 IU/L, macrosteatosis > 30%, CIT > 16 h, and WIT > 90 min), the eK^+^ concentration, the prereperfusion sK^+^ (sK^+^_0_), sK^+^_1_, and sK^+^ levels at five minutes following reperfusion (sK^+^_5_), and incidences of postreperfusion significant arrhythmias, cardiac arrest, and severe PRS.
- Data on postoperative outcomes, including re-operation and in-hospital death within the first 30 days post-LT, early allograft dysfunction (EAD), acute kidney injury (AKI), duration of mechanical ventilation, intensive care unit (ICU) admission, and length of hospital stay, were also collected.

### Study outcomes

The primary outcome was the development of PRHK, defined as the sK^+^ levels during the postreperfusion period exceeding 5.5 mmol/L. Postreperfusion significant arrhythmias and severe PRS were defined according to the Peking criteria (Table 1) [16]. AKI was diagnosed according to the Kidney Disease: Improving Global Outcomes (KDIGO) guidelines [17]. EAD was defined as meeting one or more of the following criteria: 1) aspartate aminotransferase or alanine aminotransferase levels > 2000 IU/L within the first seven days post-LT, 2) total bilirubin ≥ 10 mg/dL on postoperative day 7, or 3) an international normalized ratio ≥ 1.6 on postoperative day 7 [18].

**Table 1:** Peking criteria for the definition of severe postreperfusion syndrome in liver transplantation

Criteria	Definition	Time of diagnosis
**Significant arrhythmias**		
Bradyarrhythmia	Decrease of HR ≥ 15%	Early reperfusion period
New-onset arrhythmias	Hemodynamically significant arrhythmias (hyperkalemia-related or others)	Early reperfusion period
Cardiac arrest	Loss of spontaneous heartbeat and requires cardiac massage	Early reperfusion period
**Refractory hypotension**		
Severe hypotension	Decreased MAP unresponsive to an accumulated bolus of 1 µg/kg EP	Early reperfusion period
Persistent hypotension	Decrease of MAP ≥ 30% for ≥ 5 min regardless of the dosage of EP	Early reperfusion period
New-onset vasoplegia	NE ≥ 0.5 µg/kg/min, MAP < 50 mmHg, normal or elevated CO, and low SVR	Late reperfusion period
Prolonged vasopressor treatment	Postreperfusion hypotension requiring prolonged NE infusion to ICU	At the end of the surgery

The presence of one or more of the seven criteria indicates severe postreperfusion syndrome. *CO:* cardiac output, *EP:* epinephrine, *HR:* heart rate, *NE:* norepinephrine, *ICU:* intensive care unit, *MAP:* mean arterial pressure, *SVR:* systemic vascular resistance

### Statistical analysis

The normality of the distribution of all continuous variables was assessed using the Kolmogorov–Smirnov test. Normally distributed variables were expressed as the mean ± standard deviation, and non-normally distributed variables were expressed as the median (interquartile range). Data were compared using the Student’s *t*-tests (normal distribution) or Mann–Whitney *U* tests (non-normal distribution). Categorical variables were described as the number and percentage of patients. Data were compared using the *χ*^2^ test or Fisher’s exact test. Univariate logistic regression analyses were used to screen factors associated with the occurrence of PRHK. Potentially significant factors, which had a *P*-value < 0.10 in univariate analyses, were further enrolled in a binary logistic regression model using a forward (conditional) stepwise procedure. Receiver operator characteristic (ROC) curve analysis was used to assess the predictive accuracy and optimal cut-off value of the eK^+^ concentration. The sensitivity, specificity, and positive and negative predictive values (PPV and NPV) were determined at the optimal cut-off points. In addition, a multiple linear regression model was used to identify independent predictors of the maximum percentage increase in the sK^+^ levels following reperfusion. All statistical tests were 2-sided, and a *P*-value < 0.05 was considered statistically significant. Statistical analyses were performed using SPSS for Windows software (version 22.0; IBM SPSS, Inc., Chicago, IL, USA) and MedCalc for Windows software (version 15.2; MedCalc Software, Ostend, Belgium).

## Results

### Patients

Of 147 adults who underwent deceased LT within the study period, we excluded 55 patients without sK^+^_1_ level measurements, five patients diagnosed with hyperkalemia during the anhepatic stage, and one patient with incomplete datasets, leaving 86 patients to be included in the analyses. The clinical characteristics of the study population are listed in Table 2. The average age of the study population was 49.6 ± 10.9 years, and the majority (68.6%) were male. The leading indication for LT was hepatitis B cirrhosis (50.0%). The median (interquartile range) MELD score was 15 (9–19) points. The mean graft weight was 1302.9 ± 270.5 g, and the mean sK^+^_0_ level was 4.17 ± 0.55 mmol/L. The median (interquartile range) eK^+^ concentration was 6.65 (5.38–9.73) mmol/L.

**Table 2:** Baseline characteristics of 86 liver transplantations (LTs)

	**All LTs (*n* = 86)**	**PRHK (*n* = 54)**	**no PRHK (*n* = 32)**	***P***
Age (years)	49.6 ± 10.9	48.9 ± 11.5	50.9 ± 9.7	0.400
Male (n)	59 (68.6%)	38 (70.4%)	21 (65.6%)	0.647
Height (cm)	170 (163–175)	171 (165–175)	170 (161–175)	0.922
Weight (cm)	65.8 ± 14.7	65.8 ± 14.1	65.7 ± 16.1	0.974
Child–Pugh score	9.0 (6.0–11.0)	8.5 (6.0–11.0)	10.5 (7.0–11.8)	0.081
MELD score	15.0 (9.0–19.0)	14.0 (9.0–17.3)	16.5 (9.3–19.8)	0.312
Primary diagnosis (n)				
Hepatitis B	43 (50.0%)	22 (40.7%)	21 (65.6%)	0.026
Hepatitis C	3 (3.5%)	1 (1.9%)	2 (6.3%)	0.553
Alcoholic	11 (12.8%)	4 (7.4%)	7 (21.9%)	0.091
Cholestatic	11 (12.8%)	6 (11.1%)	5 (15.6%)	0.740
Cryptogenic	2 (2.3%)	1 (1.9%)	1 (3.1%)	1.000
Others	16 (18.6%)	7 (13.0%)	9 (28.1%)	0.081
Combined HCC	25 (29.1%)	14 (25.9%)	11 (34.4%)	0.404
Graft weight (g)	1302.9 ± 270.5	1362.7 ± 251.4	1201.9 ± 275.4	0.007
GRWR (%)	1.94 (1.61–2.43)	1.98 (1.79–2.45)	1.63 (1.43–2.28)	0.045
CIT (min)	520 (386–618)	549 (384–623)	501 (393–601)	0.381
WIT (min)	39.5 ± 6.8	38.6 ± 5.5	41.0 ± 8.4	0.143
IVC clamping time (min)	34 (31–40)	35 (32–38)	33 (30–40)	0.597
Anhepatic phase (min)	43 (38–49)	43 (37–48)	43 (39–49)	0.380
sK^+^_0_ (mmol/L)	4.17 ± 0.55	4.27 ± 0.56	4.00 ± 0.49	0.030
eK^+^ (mmol/L)	6.65 (5.38–9.73)	7.65 (5.68–12.20)	5.90 (4.40–6.88)	0.003
ECD graft (n)	13 (16.3%)	13 (24.1%)	1 (3.1)	0.011

Data are presented as mean and standard deviation (SD), median (interquartile range), or n (%). *LT:* Liver transplantation, *PRHK:* postreperfusion hyperkalemia, *MELD:* Model for End-Stage Liver Disease, *HCC:* hepatocellular carcinoma, *GRWR:* graft-to-recipient weight ratio, *CIT:* cold ischemia time, *WIT:* warm ischemia time, *IVC:* inferior vena cava, *sK*^+^_*0*_: serum potassium concentration before reperfusion, *eK*^+^: effluent potassium concentration, *ECD:* expanded criteria donor

### Postreperfusion hyperkalemia

Fifty-four (62.8%) patients experienced PRHK (Table 2). Among the primary diagnoses, only hepatitis B cirrhosis was significantly different between PRHK and non-PRHK patients (22 of 54 vs. 21 of 32, 40.7% vs. 65.6%, *P* = 0.026). Patients who developed PRHK were more often transplanted with an ECD graft (13 of 54 vs. 1 of 32, 24.1% vs. 3.1%, *P* = 0.011). Liver grafts for patients developing PRHK had a higher graft weight (1362.7 ± 251.4 vs. 1201.9 ± 275.4 g, *P* = 0.007) and a larger GRWR (1.98 [1.79–2.45] vs. 1.63 [1.43–2.28] %, *P* = 0.045) compared to patients not developing PRHK. Patients who developed PRHK had a higher sK^+^_0_ level (4.27 ± 0.56 vs. 4.00 ± 0.49 mmol/L, *P* = 0.030) and a higher eK^+^ concentration (7.65 [5.68–12.20] vs. 5.90 [4.40–6.88] mmol/L, *P* = 0.003).

### Predictors for postreperfusion hyperkalemia and the maximum percentage increase in the sK^+^ level following reperfusion

**Fig. 2:**
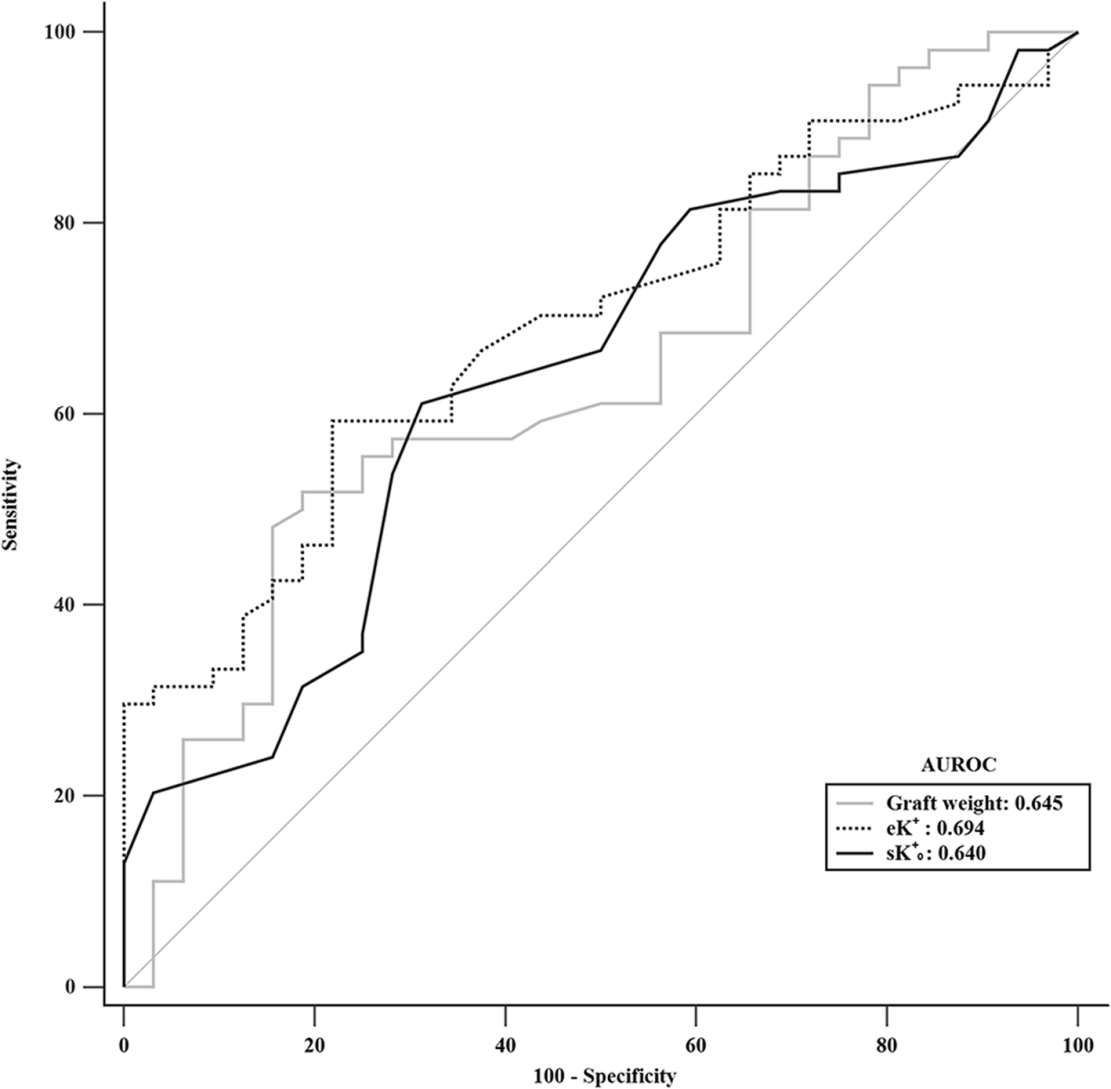
ROC curve analysis to predict the occurrence of postreperfusion hyperkalemia in 86 consecutive deceased liver transplant recipients. eK^+^, effluent potassium concentration; sK^+^_0_, serum potassium concentration before reperfusion; ROC, receiver operating characteristic

Table 3 shows an association between perioperative patient and graft variables and PRHK in patients who underwent deceased LT. After an analysis of the potentially significant predictors by multivariate logistic regression, the following three variables were independently associated with the presence of PRHK: graft weight (odds ratio [OR] 1.283; 95% confidence interval [CI] 1.029–1.599 per 100 g; *P* = 0.027), the eK^+^ concentration (OR 1.291; 95% CI 1.068–1.561 per mol/L; *P* = 0.008), and the sK^+^_0_ level (OR 4.459; 95% CI 1.543–12.884 per mol/L; *P* = 0.006). Based on the areas under the ROC curves (AUROCs), the eK^+^ concentration had the best predictive ability for the presence of PRHK (AUROC, 0.694), followed by graft weight (AUROC, 0.645) and the sK^+^_0_ level (AUROC, 0.640) (Fig. 2). Table 4 shows the sensitivity, specificity, PPV, NPV, and diagnostic accuracy at the cut-off point that provides the best Youden index for each variable. The best cut-off point for the eK^+^ concentration was more than 6.9 mmol/L, with a sensitivity of 59.26%, specificity of 78.12%, PPV of 82.05%, and NPV of 53.19%. In addition, multiple linear regression was performed to predict the maximum percentage increase in the sK^+^ level following reperfusion based on graft weight, GRWR, prereperfusion amount of RBCs transfused, the sK^+^_0_ level, the eK^+^ concentration, and the presence of an ECD graft. A significant regression equation was found (F = 10.832, *P* < 0.001), with an R2 of 0.672 (Table 5). The eK^+^ and sK^+^_0_ levels were significant predictors of the maximum percentage increase in the sK^+^ level following reperfusion.

**Table 3:** Logistic regression analysis of predictors for postreperfusion hyperkalemia in 86 consecutive deceased liver transplantation recipients

	**Univariate logistic regression**				**Multivariate logistic regression**			
	**Wald**	**OR**	**%95 CI**	***P***	**Wald**	**OR**	**%95 CI**	***P***
Age (years)	0.720	0.982	0.942–1.024	0.396				
Male (n)	0.210	0.804	0.316–2.047	0.647				
Height (cm)	0.000	1.000	0.949–1.054	0.995				
Weight (cm)	0.001	1.001	0.971–1.031	0.973				
Child–Pugh score	2.908	0.870	0.742–1.021	0.088				
MELD score	0.601	0.977	0.921–1.036	0.438				
Graft weight (100 g)	6.512	1.276	1.058–1.540	0.011	4.911	1.283	1.029–1.599	0.027
GRWR (%)	2.249	1.796	0.835–3.862	0.134				
Prereperfusion RBC transfused (units)	2.532	0.882	0.756–1.030	0.112				
Anhepatic phase (min)	0.708	0.980	0.936–1.027	0.400				
IVC clamping time (min)	0.004	0.998	0.938–1.062	0.949				
WIT (min)	2.550	0.947	0.886–1.012	0.110				
CIT (min)	0.722	1.001	0.998–1.005	0.395				
sK^+^_0_ (mmol/L)	4.515	2.536	1.075–5.983	0.034	7.627	4.459	1.543–12.844	0.006
eK^+^ (mmol/L)	8.162	1.286	1.082–1.529	0.004	6.983	1.291	1.068–1.561	0.008
ECD graft (n)	4.607	9.829	1.220–79.212	0.032				

*Abbreviations*: *CI* confidence interval, *CIT* cold ischemia time, *ECD* expanded criteria donor, *eK*^+^ effluent potassium concentration, *GRWR* graft-to-recipient weight ratio, *IVC* inferior vena cava, *MELD* Model for End-Stage Liver Disease, *OR* odds ratio, *RBC* red blood cell*, sK*^+^_*0*_ serum potassium concentration before reperfusion, *WIT* warm ischemia time

**Table 4:** Prediction of postreperfusion hyperkalemia in 86 consecutive deceased liver transplantation recipients

	**AUROC**	**Cut-off point**	**Sensitivity, *%***	**Specificity, *%***	**PPV**	**NPV**
Graft weight (100 g)	0.645	13.57	51.85	81.25	82.35	50.00
eK^+^ (mmol/L)	0.694	6.9	59.26	78.12	82.05	53.19
sK^+^_0_ (mmol/L)	0.640	4.1	61.11	68.75	76.74	51.16

*Abbreviations*: *AUROC*, area under the receiver operator characteristic curve, *eK*^+^ effluent potassium concentration, *NPV* negative predictive value, *PPV* positive predictive value, s*K*^+^_*0*_ serum potassium concentration before reperfusion

**Table 5:** Linear regression analysis of predictors for the maximum percentage increase in postreperfusion serum potassium concentration

	**B**	**SE B**	***β***	***t***	***P***
ECD graft (n)	0.084	0.070	0.122	1.200	0.234
Graft weight (100 g)	0.002	0.011	0.018	0.158	0.875
GRWR (%)	0.100	0.044	0.258	2.258	0.027
Prereperfusion RBC transfused (units)	-0.026	0.008	-0.287	-3.175	0.002
sK^+^_0_ (mmol/L)	-0.174	0.040	-0.375	-4.337	< 0.001
eK^+^ (mmol/L)	0.013	0.006	0.234	2.254	0.032

*Abbreviations*: *ECD* expanded criteria donor, *eK*^+^ effluent potassium concentration, *GRWR* graft-to-recipient weight ratio, *RBC* red blood cell*, SE* standard error, *sK*^+^_*0*_ serum potassium concentration before reperfusion

### Postreperfusion hyperkalemia and associated outcomes

Patients with PRHK had higher sK^+^_1_ (6.36 ± 0.57 vs. 4.77 ± 0.39 mmol/L, *P* < 0.001) and sK^+^_5_ (4.20 ± 0.96 vs. 3.36 ± 0.46 mmol/L, *P* < 0.001) levels, and therefore suffered more often from postreperfusion significant arrhythmias (36/54 vs. 6/32, 66.7% vs. 18.8%; *P* < 0.001) and severe PRS (43/54 vs. 18/32, 79.6% vs. 56.3%; *P* = 0.021). The occurrence of cardiac arrest after reperfusion did not differ between patients with and without PRHK. Furthermore, patients with PRHK suffered more often from postoperative EAD (32/54 vs. 5/32, 59.3% vs. 15.6%; *P* < 0.001). There were no significant differences for the other outcomes, including ventilation time, ICU and hospital stay lengths, AKI incidence, in-hospital mortality, and re-operation rate (Table 6).

**Table 6:** Comparison of postreperfusion and postoperative outcomes in patients with and without postreperfusion hyperkalemia

	**PRHK (*n* = 54)**	**no PRHK (*n* = 32)**	***P***
sK^+^_1_ (mmol/L)	6.36 ± 0.57	4.77 ± 0.39	< 0.001
sK^+^_5_ (mmol/L)	4.20 ± 0.96	3.36 ± 0.46	< 0.001
Maximum percentage increase in sK^+^ after reperfusion (%)	51.61 ± 23.62	20.27 ± 13.88	< 0.001
Significant arrhythmias (n)	36 (66.7%)	6 (18.8%)	< 0.001
Cardiac arrest (n)	6 (11.1%)	1 (3.1%)	0.250
Severe PRS (n)	43 (79.6%)	18 (56.3%)	0.021
Ventilation time (hours)	3.8 (2.7–6.6)	4.0 (2.1–5.5)	0.834
Length of ICU stay (days)	3.0 (2.5–4.0)	3.4 (2.7–4.2)	0.444
Hospitalization time (days)	18.5 (15.0–27.0)	21.0 (17.0–25.8)	0.178
EAD (n)	32 (59.3%)	5 (15.6%)	< 0.001
AKI (n)^a^	22 (43.1%)	18 (62.1%)	0.104
Re-operation (n)	4 (7.4%)	4 (12.5%)	0.432
In-hospital mortality (n)	2 (3.7%)	2 (6.3%)	0.626

Data are presented as mean and standard deviation (SD), median (interquartile range), or n (%). *AKI* acute kidney injury, *EAD* early allograft dysfunction, *ICU* intensive care unit, *PRHK* postreperfusion hyperkalemia, *PRS* postreperfusion syndrome, *sK*^+^ serum potassium concentration, *sK*^+^_*1*_ serum potassium concentration at one minute following reperfusion, *sK*^+^_*5*_ serum potassium concentration at five minutes following reperfusion. ^a^Patients with preoperative dialysis or serum creatinine ≥ 133 μmol/L were excluded

## Discussion

Elevated sK^+^ concentrations and PRHK are generally anticipated during deceased LT, especially following reperfusion of ECD liver grafts. However, little or no information is found in the literature concerning the roles of effluent fluid compositions in PRHK development. The most important finding of this study was that an elevated eK^+^ concentration was another modifiable yet potentially ignored risk factor for PRHK in deceased LT, together with the prereperfusion sK^+^ level and a large graft weight. These findings may be of significant clinical importance for PRHK prevention in deceased LT.

PRHK is associated with several intraoperative and postoperative complications, including but not limited to PRS, cardiac arrest, intraoperative death, and postoperative mortality [1–7]. Risk identification for PRHK is challenging, yet its prediction is the target of several investigations. In accordance with the present results, previous studies have demonstrated that the baseline or prereperfusion sK^+^ level was the most common modifiable factor associated with PRHK during deceased LT [8–10]. Thus, the recommendation is that when possible, greater attention and special efforts should be devoted to maintaining a relatively low sK^+^ level (less than 3.5, 4.0, or 4.5 mmol/L) prior to graft reperfusion to reduce PRHK and PRS risks [2, 9, 11, 19, 20]. Recently, Weinberg and colleagues reported that a sK^+^ level of at least 4.45 mmol/L before reperfusion is associated with PRHK [21]. In contrast, the present study had substantially more patients and demonstrated that a prereperfusion sK^+^ level greater than 4.1 mmol/L is an independent predictor of PRHK. The observed difference in the optimal cut-off point of the sK^+^_1_ level for PRHK might be attributed to center-specific differences among patient and graft characteristics.

In addition to sK^+^ levels, other predictive risk factors, such as transfusion of banked RBCs [9], metabolic acidosis during the anhepatic stage [8], and the use of ECD grafts [11–13], contribute to the development of PRHK in deceased LT. Recently, considerable attention has been focused on the relationship between graft quality and PRHK. Xia and coworkers first showed that the use of DCD grafts was independently associated with PRHK in adult LT [8]. Another study conducted by the same team confirmed that when the comparison was made with a propensity score-matched cohort of donation after brain death (DBD) grafts, DCD grafts had an increased incidence of PRHK in deceased LT [12]. Another investigation performed by Zhang et al. showed that the macrosteatotic DCD graft liver is an independent risk factor for PRHK [11]. Similar to previous reports, the present study demonstrated that the graft-dependent predictors of PRHK are graft weight and the eK^+^ concentration before reperfusion. However, a recent study showed that intraoperative PRHK did not differ between the DCD and matched DBD groups [22]. Although the authors attributed these differences to the strict criteria used for selecting liver grafts and patients, we speculate that the lack of sK^+^ level measurements during the immediate reperfusion period, which usually peaked at 30 s to one minute following reperfusion, might have affected the outcomes.

The most likely reason for the difference in the eK^+^ concentration following standard PVF is the variation in severity of hepatic ischemia–reperfusion injury (IRI) [11, 25] rather than the effect of hyperkalemic UW solution [23, 24]. The excess potassium ions in the effluent fluid may come from 1) the release of potassium from necrotic hepatocytes; 2) the passive efflux of potassium due to reduced sodium–potassium ATPase activity; or 3) the shift of potassium from hepatocytes after the exchange of hydrogen for potassium ions. Theoretically, the total amount of serum potassium is primarily determined by the patient's baseline sK^+^ level, while the abrupt release of hyperkalemic substances from the grafted liver, roughly quantified by the product of the eK^+^ concentration and the graft weight, may lead to the rise in the sK^+^ levels and the occurrence of PRHK during the immediate reperfusion period. The hypothesis above has been reversely verified in a previous study. Burlage and colleagues found that hypothermic machine perfusion, compared to traditional static cold storage, could attenuate hepatic IRI and result in a decline in sK^+^ levels or even hypokalemia following reperfusion in deceased LT [26]. An increase in perfusate potassium level, which served as an indicator similar to the eK^+^ concentration, was significantly correlated with more severe hepatic IRI.

To our knowledge, this is the first study to assess the association between hyperkalemic substances in effluent fluid and PRHK in a large LT center where ECD liver grafts are frequently encountered. Indeed, in this study, we demonstrated that the eK^+^ concentration, a simple indicator that can be obtained quickly by point-of-care blood gas analysis, was a predictive risk factor for PRHK during deceased LT. There is no standard prevention strategy for PRHK caused by a higher eK^+^ concentration or ECD liver grafts due to the lack of prospective studies. Based on our more than five years of experience, the following preventive measures can be considered: 1) the sK^+^ levels should be strictly controlled below 4.0 mmol/L; 2) graft flushing techniques, including advanced PVF with excessive flush volume [13, 24], retrograde venting via the inferior vena cava [13, 27–29], and anterograde venting via the portal vein [13, 30], can be performed to reduce the eK^+^ concentrations before reperfusion; 3) portal vein speed-control reperfusion strategy [31] should be adopted to control the timing, severity, and duration of PRHK; and 4) aggressive preemptive therapies, such as calcium chloride, epinephrine, sodium bicarbonate, and atropine, should be initiated upon reperfusion.

Several limitations are worth acknowledging. First, the retrospective, single-center design limits the study’s generalizability and may generate inevitable selection bias. Second, due to the lack of real-time, continuous monitoring methods, the sK^+^_1_ level was generally regarded as the peak sK^+^ level following reperfusion, which may underestimate the actual incidence of PRHK. Third, although multivariate analyses have demonstrated the association between the eK^+^ concentrations and PRHK, the exact mechanism of action of eK^+^ in PRHK remains to be fully elucidated. Fourth, the effectiveness of existing intervention strategies for PRHK remains unclear; therefore, further studies are warranted to address this issue definitively.

## Conclusions

A high eK^+^ concentration before reperfusion was a significant predictor of PRHK and the maximum percentage increase in the sK^+^ level following reperfusion in deceased LT. Further research is needed to evaluate whether proactive interventions can reduce the risk of PRHK during LT from a high eK^+^ concentration or ECD liver grafts.

## Data Availability

The datasets generated and/or analysed during the current study are not publicly available due to confidentiality policies but are available from the corresponding author on reasonable request.

## Abbreviations

AKI: Acute kidney injury
AUROC: Area under the receiver operating characteristic curve
CI: Confidence interval
CIT: Cold ischemia time
DBD: Donation after brain death
DCD: Donation after cardiac death
EAD: Early allograft dysfunction
ECD: Expanded criteria donor
eK^+^: Effluent potassium
GRWR: Graft-to-recipient weight ratio
ICU: Intensive care unit
IRI: Ischemia-reperfusion injury
KDIGO: Kidney Disease Improving Global Outcomes
LT: Liver transplantation
MELD: Model for End-Stage Liver Disease
NPV: Negative predictive value
OR: Odds ratio
PPV: Positive predictive value
PRHK: Postreperfusion hyperkalemia
PRS: Postreperfusion syndrome
PVF: Portal vein flush
RBCs: Red blood cells
ROC: Receiver operator characteristic
sK^+^: Serum potassium
UW: University of Wisconsin
WIT: Warm ischemia time
